# AGING IN LONG-TERM CARE FACILITIES IN BRAZIL: NEW CHALLENGES FOR INSTITUTIONAL ENVIRONMENTS

**DOI:** 10.1093/geroni/igae098.1283

**Published:** 2024-12-31

**Authors:** Sergio Luiz Tomasini

**Affiliations:** Federal University of Rio Grande do Sul, Porto Alegre, Rio Grande do Sul, Brazil

## Abstract

This contribution sheds light on the planning of outdoor spaces within Long-Term Care Facilities (LTCFs) for older person as a means to enhance the quality of life for their residents. It presents one of the few empirical studies conducted on this topic in Brazil to date. The study follows an action research approach, focusing on the participatory design of a garden for a LTCF situated in the southern region of the country. The project unfolded in three stages: 1) Initial interviews were conducted with residents to explore their utilization of the institution’s external areas, their past experiences with gardens and their perceptions of the “ideal garden.”. Additionally, systematic observations were made regarding how residents interacted with the open spaces within the LTCF; 2) Subsequent collaborative meetings aimed to actively involve residents in selecting the garden site and defining project requirements; 3) Finally, interviews were held with participating residents to assess the effectiveness of the project methodology. The analysis of these interviews utilized the meaning condensation method, leading to the identification of several themes related to the participatory project approach: a) possibility of creating a territory in the facility; b) opportunity to develop an active role in the facility; c) acquisition of new knowledge; d) promotion of social relations. Overall, the residents’ feedback suggests that employing participatory planning methods can positively impact the adaptive outcomes of older adults in institutional settings.

